# Supplementary material for: Field-Crop Soils in Eastern France: Coldspots of Azole-Resistant Aspergillus fumigatus
Source: J Fungi (Basel). 2023 May 27;9(6):618. doi: 10.3390/jof9060618 (PMC10301667; doi:10.3390/jof9060618)
Supplement: Supplementary file 1 [file jof-09-00618-s001.zip › jof-2346211-supplementary.pdf]

SUPPLEMENTAL DATA

	Precursor ion	Production ion	Collision energy (eV)	Retention time (min)
Propiconazole	173	146	18	26.361; 27.066
	173	109	30	
Tebuconazole	125	99	20	27.353
	125	89	20	
Epoxyconazole	192	138	12	26.648; 27.918
	192	111	32	
Bromuconazole	173	145	26	28.312; 29.016
	173	109	30	
Difenconazole	265	202	36	34.366; 34.485
	265	139	40	
Tebuconazole d6	256	125	26	27.287
	256	73	10	

Table S1: Mass spectrometry parameters of the triazoles studied

	Isolates	Plot ID	Culture media of strain detection	MIC voriconazole (mg/L)	MIC itraconazole (mg.L)
BURGUNDY	1	POP	Malt + itraconazole	1	0.25
	2	POP	DG18	0.5	0.5
	3	POP	DG18	0.25	0.25
	4	POP	DG18	0.5	0.5
	5	POP	DG18	0.5	0.25
	6	MJP	DG18	1	0.5
	7	MJP	DG18	1	0.5
	8	MJP	DG18	0.5	0.5
	9	MJP	DG18	0.5	0.5
	10	CHP	DG18	0.5	0.25
	11	MOP	Malt + voriconazole	1	0.5
	12	MOP	Malt + voriconazole	1	0.5
	13	MOP	Malt + voriconazole	1	1
	14	MOP	Malt + voriconazole	1	0.5
	15	MOP	Malt + voriconazole	1	0.5
	16	MOP	Malt + voriconazole	1	0.5
	17	MOP	Malt + voriconazole	0.5	1
	18	MOP	Malt + voriconazole	1	0.5
	19	MOP	Malt + voriconazole	1	0.5
	20	MOP	Malt + voriconazole	1	0.5
	21	MOP	Malt + voriconazole	1	1
	22	MOP	Malt + voriconazole	0.5	1
	23	MOP	Malt + voriconazole	1	0.5
	24	MOP	Malt + voriconazole	1	0.5
	25	MOP	Malt + itraconazole	1	0.5
	26	MOP	Malt + itraconazole	1	0.5
	27	MOP	Malt + itraconazole	1	0.5
	28	MOP	Malt + itraconazole	1	0.5
	29	MOP	Malt + itraconazole	1	0.5
	30	MOP	Malt + itraconazole	1	0.5
	31	MOP	Malt + itraconazole	1	0.5
	32	MOP	DG18	0.5	0.5
	33	MOP	DG18	0.5	0.25
	34	MOP	DG18	0.5	0.5
	35	MOP	DG18	1	0.5
	36	MOP	DG18	1	0.5
	37	QUP	DG18	0.5	0.25

38	QUP	DG18	0.5	0.5
39	QUP	DG18	0.5	0.5
40	QUP	DG18	0.5	1
41	QUP	DG18	0.5	1
42	QUP	DG18	1	0.5
43	MAP	DG18	0.5	0.25
44	MAP	DG18	0.5	0.5
45	MAP	DG18	0.5	0.5
46	BGP	Malt + itraconazole	1	0.5
47	BGP	Malt + itraconazole	1	0.5
48	BGP	Malt + itraconazole	1	0.5
49	SFP	Malt + voriconazole	1	0.5
50	SFP	Malt + voriconazole	0.5	0.5
51	SFP	Malt + voriconazole	1	0.5
52	SFP	Malt + voriconazole	1	0.5
53	SFP	Malt + voriconazole	1	0.5
54	SFP	Malt + voriconazole	1	0.5
55	SFP	Malt + voriconazole	1	1
56	SFP	Malt + voriconazole	1	0.5
57	SFP	Malt + voriconazole	1	1
58	SFP	Malt + itraconazole	1	0.5
59	SFP	Malt + itraconazole	1	0.5
60	SFP	Malt + itraconazole	1	0.5
61	SFP	Malt + itraconazole	1	0.5
62	SFP	Malt + itraconazole	1	1
63	SFP	DG18	1	1
64	SFP	DG18	0.5	0.5
65	SFP	DG18	0.5	0.5
66	SFP	DG18	1	0.5
67	SFP	DG18	0.5	0.5
68	SFP	DG18	0.5	0.25
69	SFP	DG18	0.5	0.5
70	SFP	DG18	1	0.5
71	SFP	DG18	0.5	0.5
72	SFP	DG18	1	0.5
73	SFP	DG18	0.5	0.5
74	SFP	DG18	0.5	0.25
75	SFP	DG18	0.5	0.25
76	SFP	DG18	1	0.5

	77	SFP	DG18	0.5	0.5
	78	SFP	DG18	0.5	0.5
	79	SFP	DG18	1	0.5
	80	SFP	DG18	0.5	0.5
	81	SFP	DG18	0.5	0.5
	82	SFP	DG18	1	0.5
	83	SFP	DG18	0.5	0.25
	84	SFP	DG18	0.5	1
	85	LHP	DG18	0.5	0.25
	86	LHP	DG18	0.5	1
	87	LHP	DG18	0.5	0.5
	88	LHP	DG18	0.5	0.5
	89	LHP	DG18	0.5	0.5
	90	LHP	DG18	0.5	0.25
	91	LHP	DG18	0.5	0.5
	92	LHP	DG18	0.5	0.5
	93	LHP	DG18	1	0.5
	94	LHP	DG18	1	0.5
	95	LHP	DG18	0.5	0.5
	96	LHP	DG18	1	0.5
	97	LHP	DG18	0.5	0.5
	98	LHP	DG18	0.5	0.5
	99	LHP	DG18	1	0.25
	100	LHP	DG18	0.5	0.5
	101	LHP	DG18	1	0.5
LORRAINE	102	BP	Malt + itraconazole	0.5	0.25
	103	PCP	Malt + itraconazole	1	1
	104	RP	Malt + itraconazole	1	0.5
	105	DJP	Malt + itraconazole	0.5	1
	106	DJP	Malt + itraconazole	0.5	1
	107	DJP	Malt + itraconazole	0.5	0.5
	108	DJP	Malt + itraconazole	1	1
	109	DJP	Malt + itraconazole	1	0.5
	110	DJP	DG18	0.25	0.25
	111	DJP	DG18	1	0.25
	112	DJP	DG18	0.25	0.5
	113	DJP	DG18	0.5	1
	114	DJP	DG18	0.5	0.5
	115	DJP	DG18	0.5	1

	116	DJP	DG18	0.5	0.5
	117	DJP	DG18	1	0.5
	118	DJP	DG18	0.5	1
	119	DJP	DG18	0.5	0.5
	120	DJP	DG18	1	1
	121	DJP	DG18	0.5	1
	122	DJP	DG18	0.5	1
	123	DJP	DG18	0.25	0.25
	124	DJP	DG18	0.5	0.5
	125	DJP	DG18	1	0.25
	126	DJP	DG18	1	0.5
	127	DJP	DG18	0.5	0.5
	128	DJP	DG18	0.25	0.5
	129	DJP	DG18	0.5	0.5
	130	DJP	DG18	0.5	0.5
	131	DJP	DG18	0.5	0.125
	132	DJP	DG18	0.25	0.25
	133	VSP	Malt + itraconazole	0.5	0.25
	134	VSP	Malt + itraconazole	0.5	0.5
	135	VSP	Malt + itraconazole	0.5	1
	136	VSP	Malt + itraconazole	1	0.5
	137	VSP	Malt + itraconazole	0.5	0.25
	138	VSP	DG18	0.5	0.125
	139	VSP	DG18	0.5	0.125
	140	VSP	DG18	0.25	0.5
	141	VSP	DG18	1	0.5
	142	VSP	DG18	1	0.5
	143	VSP	DG18	1	0.25
	144	VSP	DG18	0.5	0.25
	145	VSP	DG18	0.5	1
	146	VSP	DG18	0.25	0.5
	147	VSP	DG18	1	1
	148	VSP	DG18	1	0.5
	149	VSP	DG18	0.5	0.25
	150	VSP	DG18	0.5	0.5
	151	VSP	DG18	0.5	0.5
	152	VSP	DG18	0.5	0.25
	153	VSP	DG18	1	0.5
	154	VSP	DG18	1	0.5
	155	TP	Malt + itraconazole	1	0.5

	156	TP	Malt + itraconazole	0.5	0.5
	157	TP	Malt + itraconazole	1	0.5
	158	TP	Malt + itraconazole	1	0.5
	159	TP	Malt + itraconazole	1	0.5
	160	TP	DG18	0.5	0.25
	161	TP	DG18	0.5	0.5
	162	TP	DG18	0.5	0.5
	163	TP	DG18	1	0.5
	164	GCP	Malt + itraconazole	1	1
	165	LIP	Malt + itraconazole	1	1
	166	LIP	Malt + itraconazole	1	0.5

Table S2: Characteristics of isolates: media of detection and minimal inhibition concentrations to itraconazole and voriconazole.

Target Name	Quantity (fg/µL)	Cq Mean	Cq SD	Amplification efficiencies	R^2
A fumigatus	5000	25.9	0.2		
A fumigatus	500	29.6	0.2		
A fumigatus	50	33.0	0.1	90.2%	0.989
A fumigatus	5	36.8	0.6		
A fumigatus	0.5	40.2	1.2		
Panfungal	5000	26.6	0.3		
Panfungal	500	30.4	0.3		
Panfungal	50	34.0	0.4	91.7%	0.988
Panfungal	5	37.6	0.4		
Panfungal	0.5	40.6	1.1		

Table S3: Cq obtained, amplification efficiencies and the R^2 for qPCR assays (standard curves)

		Response variables		
Explanatory variables	Test	*A. fumigatus* isolated by culture	*A. fumigatus* quantified by qPCR	Fungal load quantified by qPCR
Region	Wilcoxon	0.26	0.24	0.15
Fungicide treatment	Kruskal Wallis	0.58	0.26	0.61
Type of culture	Kruskal Wallis	0.58	0.59	0.26
Organic matter content	Spearman correlation	0.38	0.19	0.90
pH	Spearman correlation	0.53	0.31	0.39
Proportion of silt	Spearman correlation	0.99	0.41	0.75
Proportion of clay	Spearman correlation	0.66	0.88	0.22
Proportion of sand	Spearman correlation	0.82	0.79	0.51

Table S4: Outputs of statistical analysis (p-values).
